# Strange relatives: the enigmatic arbo-jingmenviruses and orthoflaviviruses

**DOI:** 10.1038/s44298-025-00106-z

**Published:** 2025-04-04

**Authors:** Edwin O. Ogola, Amitava Roy, Kurt Wollenberg, Missiani Ochwoto, Marshall E. Bloom

**Affiliations:** 1 Biology of Vector Borne Viruses Section, Laboratory of Virology, Rocky Mountain Laboratories, NIAID, NIH, 903 South 4th Street, Hamilton, MT 59840 USA; 2 Department of Biomedical and Pharmaceutical Sciences, University of Montana, 32 Campus Drive, Missoula, MT 59812 USA; 3 Bioinformatics and Computational Biosciences Branch, Office of Cyber Infrastructure and Computational Biology, NIAID, NIH, 31 Center Drive, Room 3B62, Bethesda, MD 20892-0485 USA

**Keywords:** Viral evolution, Viral reservoirs, Viral transmission, Viral vectors, Virus-host interactions

## Abstract

Arthropod - and vertebrate-associated jingmenviruses (arbo-JMV) have segmented positive-strand RNA genomes and are provisional members of the genus *Orthoflavivirus* (family *Flaviviridae*). Current investigations have described arbo-JMV infection in vertebrate hosts in proximity to humans. This raises concerns about the virus host range and public health implications. This review explores the genomic and evolutionary relationship between arbo-JMV and orthoflaviviruses and evaluates the potential of arbo-JMV to pose a public health threat.

## Introduction

Jingmenviruses (JMV) are a group of rapidly emerging viruses. They were first described in *Rhipicephalus microplus* ticks collected in 2010 near Jingmen City, China, during a survey for Huaiyangshan virus^1^. Currently, JMV has been identified in continental Asia, Africa, Oceania, Europe, North and South America^2–10^. The JMV are regarded as provisional members of the genus *Orthoflavivirus* (family *Flaviviridae*)^11,12^, largely because of their segmented genome structure and protein function^1,13^. Other orthoflaviviruses like West Nile virus (WNV), dengue virus (DENV) and Powassan virus/deer tick virus (POWV/DTV) are able to infect a wide variety of animal hosts and are associated with serious health consequences in human^14,15^.

The JMV genome is comprised of single-stranded, positive-sense RNA (ss (+) RNA) encoding structural and non-structural proteins (NS), similar to the orthoflaviviruses genome. However, unlike other orthoflaviviruses, the genome is in 4 or 5 segments; each containing at least one open reading frame and each flanked by 5’ and 3’untranslated regions (UTRs)^16–19^. Segments 1 and 3 contain sequences, respectively, homologous to orthoflavivirus NS5 and NS3-NS2B complex, while segments S2 and S4 are highly divergent and bear no obvious sequence similarity to known viral proteins^1^. Segment 5 is non-essential during virus replication and has so far been associated with only insect-restricted JMV, including Guaico Culex virus (GCXV) and Mole Culex virus (MoCV)^17,20,21^. The description of GCXV genomic segments represent the multipartitism nature of JMV genome, however, the mechanism and impact of this genome architecture on viral replication remains undetermined^17,20–22^. Nevertheless, the characteristic genome organization pattern provides an important insight into possible relationships with other orthoflaviviruses^23^, highlighting our limited understanding of the prevalence and evolution of these pervasive viral agents.

**Fig. 1:**
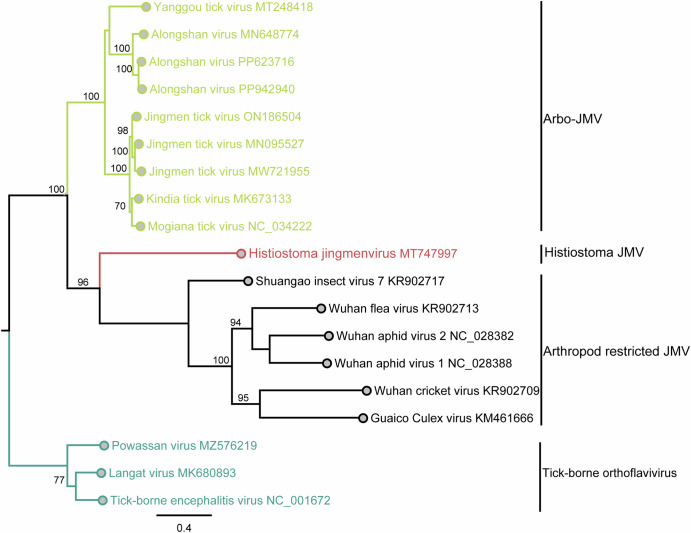
Phylogenetic tree for jingmenviruses (JMV) in the *orthoflaviviruses* group inferred by maximum-likelihood analysis in PhyML v. 2.2.4 and assessed over 1000 bootstraps. The phylogenetic tree based on JMV Segment 1 comprises arbo-JMV group infecting both arthropods and vertebrates, arthropod-restricted group, *Histiostoma jingmenvirus* (HJMV) group and tick-borne orthoflaviviruses.

Another similarity between JMV and more typical orthoflaviviruses is the host range. By host preference range, the orthoflaviviruses are clustered into three broad phylogenetic groups (a) the no-known-vector flaviviruses (NKV) with no established vector involved in their transmission, (b) the vertebrate pathogenic group containing the mosquito- and tick-borne orthoflaviviruses, and (c) the arthropod-specific group which reproduces exclusively in arthropods and lacks any defined ability to infect vertebrates^24–26^. At present, the JMV can be broadly categorized into three broad phylogenetic groups (Fig. 1): (a) an arbo-JMV group that infects both arthropods and vertebrates represented by *Jingmen tick virus* (JMTV), *Alongshan virus* (ALSV), *Mogiana tick virus* (MGTV), *Kindia tick virus* (KITV) and Yanggou tick virus (YGTV)^20^, (b) an arthropod restricted group such as GCXV, MoCV, *Carajing virus* (CaJV), *Inopus flavus jingmenvirus* 1 (IFJV1) among others^20^ and (c) the *Histiostoma jingmenvirus* (HJMV) group, which clusters between arbo- and arthropod-associated JMV. This latter group has been identified in mites collected from home environments but has so far not been detected in an established vector or vertebrate^27^.

The proper containment level of new viruses is determined by standard processes at most institutions and many orthoflaviviruses, such as POWV, Japanese encephalitis virus (JEV) and Louping ill virus (LIV), are classified as biosafety level 3 (BSL-3). However, the majority of the JMVs remain unclassified apart from the BSL-2 GCXV (https://www.cdc.gov/labs/BMBL.html). As additional information is acquired on the potential for these viruses to cause human and animal disease, it is very likely that more of the JMV will be formally classified.

The potential pathogenicity of the arbo-JMV for humans has been demonstrated by antibodies and by isolation of viable JMTV and ALSV from patients with a history of tick bite^2,28,29^. To date, there are no reports of vertebrate mortality associated with arbo-JMV infection; however, available data suggest that humans experience unique clinical symptoms. In JMTV and ALSV infection, the most common clinical symptoms in the patients include headache and fever^2,28,29^. In vitro experimental infections demonstrate that arbo-JMV can infect diverse mammalian cells^30,31^, however, pathogenicity for animals other than human and mice remains undetermined. Although reverse zoonosis transmission is still unclear, arbo-JMV zoonotic potential has been established by the detection of viral RNA and antibodies in animals as well as parasitizing ticks^32–35^. Furthermore, evidence of circulation within communities has been shown by the existence of JMTV RNA in wastewater and environmental samples^36,37^. Importantly, there is as yet no report of direct spread between humans. Actual isolations of the virus are rare, but arbo-JMV viral nucleic acids have been detected in a variety of animals in close proximity to humans. These include arthropods, reptiles and mammals, including cattle, sheep, goats and horses, all of which accentuate concerns about the public health significance and host range of arbo-JMV^2,17,28,32,38^.

Considering the diverse potential hosts, it is likely that many arbo-JMV remain undiscovered in various ecologies. Therefore, this review summarizes existing information on the arbo-JMV discovery and genome organization. Further, to provide insight into arbo-JMV transmission dynamics, the review seeks to address questions such as what is the host range of arbo-JMV? Do arbo-JMV have specific animal host reservoirs? Is there an optimal cell line for isolation and stable growth of arbo-JMV? In addition, is there evidence of arbo-JMV onward spillover?

Because of the concerns about host range and public health significance, we review investigations focusing on JMTV and ALSV, both of which have been associated with human illness. In addition, we propose the development of arbo-JMV animal models to improve understanding of transmission dynamics. Our key message is the need to investigate newly emerging arboviruses and evaluate their potential public health threats.

## Isolation and geographic distribution of arbo-JMV

**Fig. 2:**
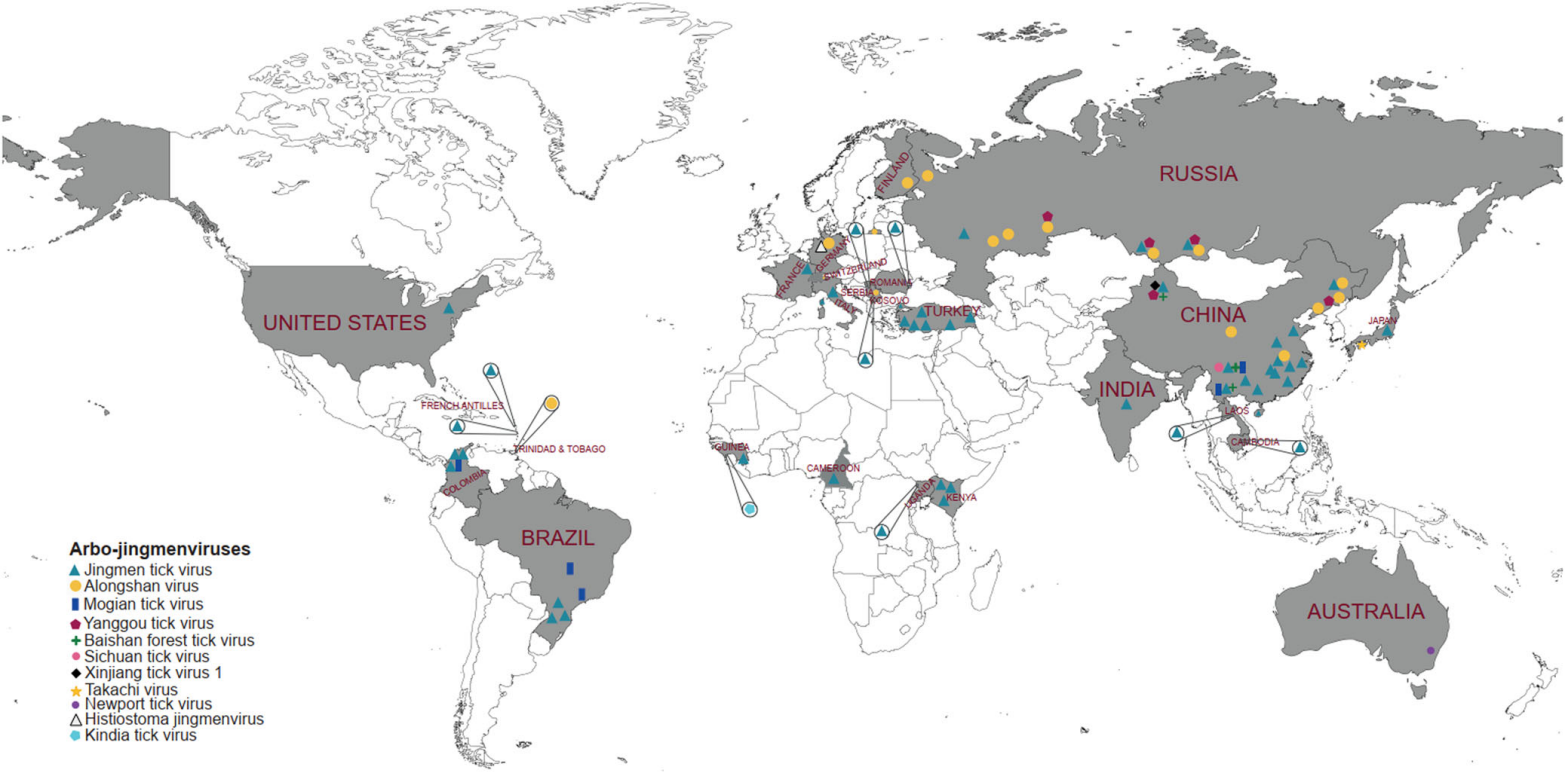
Geographic distribution of arthropod - and vertebrate-associated jingmenviruses. The arbo-JMV viruses have been detected worldwide; more genetically diverse strains have been observed in China and Russia than in any other geographic region. The map was created in https://mapchart.net/world.html and modified by authors.

The first evidence of JMV was RNA of Jingmen tick virus (JMTV) identified in *Rhipicephalus microplus* ticks collected in the Jingmen region of China in 2010^1^. JMTV was later isolated from *Amblyomma javanense* ticks collected from pangolins in China and from *R. bursa* ticks collected from sheep in Turkey ^2,3^. Alongshan virus (ALSV) was isolated from *Ixodes persulcatus* ticks collected in Russia^39,40^. Another arbo-JMV, YGTV, was isolated from *Dermacentor reticulatus*, *D. marginatus*, *I. persulcatus* and *D. nuttalli* ticks collected in Russia^39,41,42^. Current evidence indicates that arbo-JMV are present world-wide as depicted in Fig. 2 and detailed in Supplementary Table 1, the geographic distribution of arbo-JMV isolates is also summarized in Table 1. In parts of Asia and Europe arbo-JMV distribution overlaps with that of other tick-borne orthoflaviviruses such as tick-borne encephalitis virus (TBEV) complex^43,44^. In these regions, *I. ricinus* and *I. persulcatus* are known vectors of TBEV. In areas without Ixodid ticks, TBEV transmission relies on ticks of *Haemaphysalis* and *Dermacentor* species^43,44^, potentially sharing vector species with arbo-JMV (Supplementary Table 1). Similarly, both arbo-JMV and TBEV are capable of infecting the same vertebrate host, as shown by the detection of viral RNA and antibodies in livestock and deer^43^. The distribution overlap of arbo-JMV and other tick-borne orthoflaviviruses and the association with the same vector species and vertebrate host implies that arbo-JMV origin and the segmented genomic structure may be a result of genetic recombination and reassortment during co-infection with other tick-borne orthoflaviviruses^1,45^. However, the exact genetic recombination events and evolutionary patterns remain undetermined.

**Table 1:** Geographic distribution of actual arbo-jingmenviruses isolates

Arbo-jingmenvirus (Abbr.)	Tick and mosquito species	Vertebrate host	Isolated in cell culture	Cell Line	CPE	Antibodies detected	Region	Country	Reference
Jingmen tick virus (JMTV)	*A. javanense, I. persulcatus*	Human, Pangolins	Yes	BME/CTVM23		Human	Guangxi	China	^2^
	*R. microplus*	Cattle	Yes	BME/CTVM23, IRE/CTVM19	No	No	Loudi	China	^82^
	*R. bursa, R. turanicus, Hy. marginatum, H. parva, R. sanguineus* sensu lato *H. inermis*	Dog, Sheep, Goat, Cattle	Yes	Vero E6	Yes	No	Van, Mersin, Kirklareli, Tekirdag	Turkey	^3^
Alongshan virus (ALSV)	*I. persulcatus*	Unknown^a^	Yes	HAE/CTVM8	No	No	Chelyabinsk	Russia	^42^
	*I. persulcatus*	Unknown^a^	Yes	IRE/CTVM19	No	No	Karelia Chelyabinsk	Russia	^40^
	*D. reticulatus, I. ricinus, H. concinna, D. nuttalli, I. persulcatus*	Unknown^a^	Yes	IRE/CTVM19, HAE/CTVM8	No	No	Ulyanovsk, Tatarstan, Tuva, Kaliningrad, Altai, Chelyabinsk Karelia,	Russia	^39^
	*I. persulcatus, Cx*. *tritaeniorhynchus, An. yatsushiroensis*	Human	Yes	Vero, Hepa 1-6 BHK-21, U-87MG, HFF	Yes	Yes	Mongolia, Heilongjiang, Jilin	China	^28^
	Unknown^b^	Sheep, Cattle	Yes	Vero	Yes	Yes	Mongolia	China	^32^
Yanggou tick virus (YGTV)	*D. reticulatus, D. marginatus, I. persulcatus*	Unknown‡	Yes	HAE/CTVM8	No	No	Chelyabinsk	Russia	^42^
	*D. nuttalli, D. marginatus*	Cattle	Yes	IRE/CTVM19, HAE/CTVM8	No	No	Tuva, Altai	Russia	^39^

*R.* Rhipicephalus, *A.* Amblyomma, *I.* Ixodes, *H.* Haemaphysalis, *D.* Dermacentor, *Hy.* Hyalomma, *Cx.* *Culex*, *An. Anopheles*, *BME/CTVM23* Rhipicephalus microplus origin cells, Vero *Chlorocebus sabaeus* (Green monkey) origin cells, *HAE/CTVM8* Hyalomma anatolicum anatolicum origin cells, *IRE/CTVM19* Ixodes ricinus origin cells, *Hepa 1-6* Mus musculus, *BHK-21Mesocricetus auratus* origin cells, *U-87MG* Homo sapiens origin cells, HFF *Homo sapiens* origin cells, *CPE* cytopathic effect.

^a^Ticks collected by flagging.

^b^Tick species not surveyed.

## Genome structure and function

**Fig. 3:**
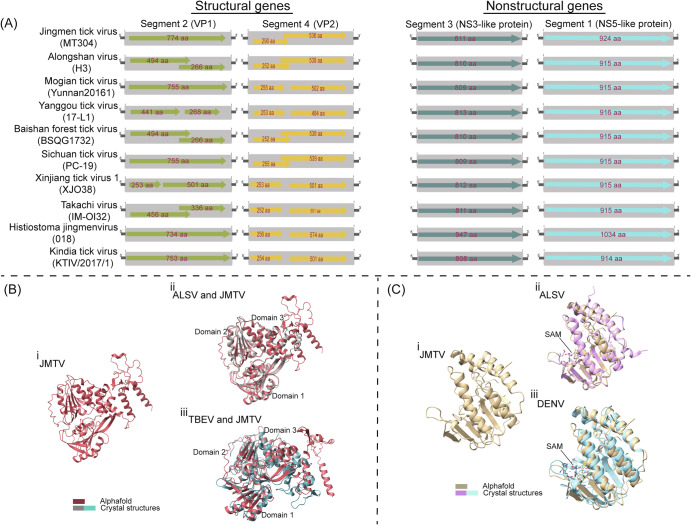
Genomic organization and function of arthropod- and vertebrate-associated members (arbo-JMV). **A** Genomic organization of arbo-JMV. The coding region and translational direction are indicated by means of a different colour for each segment. The virus strain is indicated in the bracket. Segments 1 and 3 in all arbo-JMV code one protein, while segments 2 and 4 code for either one or two proteins depending on the virus strain. **B** Jingmen tick virus (JMTV), Alongshan virus (ALSV) and Tick-borne encephalitis virus (TBEV) NS3 helicase structural models. **i** JMTV NS3 helicase structure predicted by Alphafold. **ii** JMTV NS3 helicase structure superimposed onto experimentally determined ALSV NS3 helicase structure (PDB code: 6M40)^49^. **iii** JMTV NS3 helicase structure superimposed onto experimentally determined TBEV NS3 helicase (PDB code: 7OJ4)^51^. The functional domains are shown in **ii** and **iii**. **C** JMTV, ALSV and dengue virus methyltransferase (MTase) structural models. **i** JMTV MTase structure predicted by Alphafold. **ii** JMTV MTase structure superimpositioned onto experimentally determined ALSV MTase structure (PDB code: 8GY9)^55^. **iii** JMTV MTase structure superimpositioned onto experimentally determined Dengue virus MTase structure (PDB code: 3P97)^83^. The position of S-adenosyl-l-methionine (SAM) methyl donor during viral cap formation is shown in **ii** and **iii**.

Advanced nucleic acid sequencing and analytical techniques have enabled the characterization of the arbo-JMV genome structure and organization, sometimes even in the absence of actual virus isolates^1,2,28,29,46^. Thus, full genome sequences of several arbo-JMV have been obtained and others such as Newport tick virus (NTV) are partially characterized^47^. The genomes of the arbo-JMV resemble those of orthoflaviviruses. In every case, the results indicate a multipartite genome with four segments, arbitrarily denoted as S1–S4^2,17,28,29^. Based on the available data, the complete genome is most likely single-stranded, positive-sense RNA (ss (+) RNA) encoding both structural and non-structural (NS) proteins^1^. Because the length of each segment ranges from 2800 bp to 3700 nucleotides, the full genome size exceeds the 11 kb size of a typical orthoflavivirus (Fig. 3A).

Each segment has a putative coding region for at least one protein and is flanked by both 5’ and 3’ untranslated regions (UTRs), findings that support a bonafide segmented genome. There is sequence conservation at the 5’- and 3’-UTRs among the segments^16–19^. There is a conserved 5’-CAAGUG-3’ 3’-UTR sequence in all the segments of ALSV and JMTV^16–18^. As with typical orthoflaviviruses, these conserved sequences are likely involved in the RNA structure formation and virus replication. In contrast, sequence differences in the 5’ UTRs across members of the JMV group might reflect differences in the replication processes or host tropism^18^.

## Nonstructural protein similarities

The open reading frames (ORF) of 2 segments have homology to orthoflavivirus NS proteins. The orthoflavivirus NS3 has protease and helicase activities crucial in virus replication, RNA polyprotein processing, and viral cap formation, respectively^48^. Segment 3 (S3) ORF codes for non-structural protein 2 (NSP2) homologous to the orthoflavivirus NS3-NS2B complex both in the N-terminal helicase portion and the C-terminal protease^1^. NS3-NS2B complex structure and function are well studied in orthoflaviviruses but, with the exception of ALSV remain less investigated in arbo-JMV. Biochemical and biophysical experiments show that ALSV NS3-like helicase ATPase activity and overall folding are comparable to those of orthoflaviviruses^49^. These findings are further illustrated by the similarity of folding among structure models of JMTV, generated by the artificial intelligence-based algorithm AlphaFold^50^, and crystal structure comparisons of ALSV (root mean square deviation, RMSD 1.002 Å) and tick-borne encephalitis virus (TBEV, RMSD 1.137 Å) (Fig. 3B)^49,51^. However, in spite of these shared topological features, arbo-JMV and orthoflaviviruses NS3 helicase have low sequence similarities, 15–28%, between themselves^49^.

Arbo-JMV segment 1 (S1) ORF shows homology to the orthoflavivirus NS5, which contains the RNA-dependent RNA polymerase (RdRp) and methyltransferase (MTase) activities^1,52^. The RdRp of arbo-JMV and the more typical orthoflaviviruses have topological similarity and superimpose well on each other, indicating similar functions in virus replication^53,54^. Crystal structures of ALSV MTases show great structural similarity with its *Orthoflavivirus* homologues^55^, but the MTase gene structure and activity are uninvestigated for other arbo-JMV. Nevertheless, although a comparison of arbo-JMV MTases shows very low amino acid sequence identity with closely related orthoflaviruses, 15–28% (Supplementary Fig. 1), similarities are further supported by AlphaFold generated models of JMTV and crystal structure comparisons of ALSV (RMSD 0.671 Å) and dengue virus (DENV, RMSD 1.133 Å) (Fig. 3C)^50,55^.

The comparability of these 2 arbo-JMV and orthoflavivirus NS protein structures (RMSD 1.133 and 1.137 Å) in the face of low sequence similarity suggests that promising antiviral compounds targeting orthoflaviviruses NS proteins may not show comparable activity against arbo-JMV NS proteins. Therefore, arbo-JMV structures should be included when modelling new drug compounds against orthoflaviviruses and related emerging infectious diseases.

## Structural proteins divergence

The coding sequences for arbo-JMV S2 and S4 show no similarity to known orthoflaviviruses in protein sequence databases^1^. Computational analysis of arbo-JMV S2 amino acid sequences predicts that protein is a capsid and membrane protein^56^. However, in spite of the fact that the S2 arbo-JMV proteins have substantial amino acid similarities to membrane proteins with multiple transmembrane domains, there is no similarity to the structural proteins of other orthoflaviviruses^56^.

Similarly, S4 is suspected to encode envelope glycoproteins referred to as VP2 and VP3^1^. Once again, arbo-JMV glycoproteins share substantial amino acid similarities among themselves but lack similarity to the glycoproteins of other orthoflaviviruses. Nevertheless, prediction suggests that arbo-JMV glycoproteins have traits associated with class II viral fusion proteins important for virus entry into host cells^56^. Although S2 and S4 have been shown to be related to *Toxocara canis* larval cDNA library transcripts, it is intriguing that S2 and S4 have definite features of viral structural proteins but fail to resemble other viruses^1^. Clearly, much study is needed.

## 5’ and 3’untranslated regions (UTRs) sequence conservation similarities

All the four genome segments have 5’ and 3’ UTRs and there is sequence conservation in 5’ and 3’ among the segments (Fig. 3A)^1,18^. In the 3’ UTR, ALSV and JMTV have conserved 5’-CAAGUG-3’ sequences in all the segments^18,19^. By analogy with typical orthoflaviviruses, the conserved sequences are likely involved in the RNA structure formation and regulation of virus replication^57^. In contrast, there are differences in the 5’ UTR conserved sequences across members of the JMV group, perhaps reflecting differences in the replication processes and host tropism^18^. In addition, in other arbo-JMV such as KITV, 5’ and 3’ UTRs modelling and functional annotation revealed the presence of viral replication and translation regulatory elements such as multiple UAG sites and 5’/3’ downstream AUG region (DAR), which have also been described in orthoflaviviruses^19,58,59^. In contrast to orthoflaviviruses where 3′ UTR structure facilitates the formation of subgenomic flavivirus RNA (sfRNA) attributed to host immune evasion and pathogenicity, the structuring and function of sfRNA in arbo-JMV remain uninvestigated^18,60–62^.

## Polymerase gene integration similarities

Another similarity between arbo-JMV and orthoflaviviruses is the integration of the RdRp gene into the invertebrate host genome. For example, the study of Morozkin et al. demonstrate the integration of JMTV and ALV RdRp gene into *I. ricinus* genome^63^. The observed virus-derived DNA integration in the host genome is so far limited to vectors and has also been demonstrated by the presence of orthoflaviviruses-like sequences of Cell Fusing Agent virus (CFAV) and Kamiti River virus (KRV) in *Aedes* mosquitoes^64–66^. While in mosquitoes, the significance of orthoflaviviruses-derived gene integration in the host genome has been associated with improved tolerance to viral infection and survival^67^, the importance of arbo-JMV gene integration is undetermined in ticks.

## Evolutionary lineage

Evolutionary virology analyses have identified endogenous viral elements (EVES) homologous to arbo-JMV suggesting that orthoflaviviruses are ancient by hundreds of years and that arbo-JMTV originated several years ago^68^. The identification of arbo-JMTV genome organization and the ancestral relationship with unsegmented orthoflaviviruses represent a rare occurrence in virus emergence and evolution^1^. Although the exact mechanism remains undescribed, it is suspected that arbo-JMV emerged from unsegmented orthoflavivirus and an unrelated virus coinfecting the same host, which resulted in recombination and reassortment of structural and nonstructural proteins as observed in RNA-DNA hybrid virus^1,69^. The impact of this genomic structuring is poorly described. Computational analyses suggest that arbo-JMV genome organization into shorter segments might improve virion stability^70,71^. Further, the genomic packaging enhances arbo-JMV genetic recombination and reassortment, leading to the emergence of new viral strains^72^. However, genomic components of segmented genomes might have different infection rates during host invasion lowering successful infection^73^. These findings may be supported by arbo-JMV maintenance in diverse hosts and the challenge of stably growing arbo-JMV in cell culture.

**Fig. 4:**
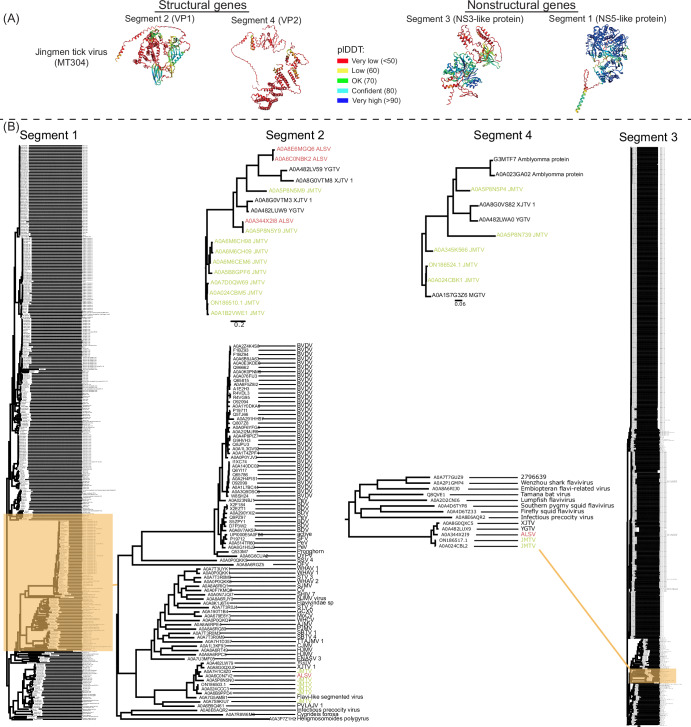
Evolutionary lineage of arthropod- and vertebrate-associated members (arbo-JMV). **A** Jingmen tick virus (JMTV) AlphaFold predicted protein structures. The colours indicate confidence scores shown by the predicted local distance difference test (pLDDT). **B** The maximum-likelihood phylogenetic trees inferred by PhyML v. 2.2.4-embedded in Geneious prime with tree reliability assessed over 1000 bootstraps.

Protein evolution and time-direct molecular clock analysis complement each other. Beyond evolution details, protein evolutionary analysis provides additional information such as protein function. Several arbo-JMV genome sequences are available through public databases and recent studies have reconstructed time-trees demonstrating arbo-JMV evolutionary relationships and potential genetic recombination during virus transmission^45^. However, arbo-JMV protein structures and functions are less studied. To track the evolutionary lineage of arbo-JMV, Alphafold was used to predict structural models of JMTV proteins, including until now S2 and S4 structural models (Fig. 4A)^50^. The predicted protein structures were comparable to those obtained using EMSFold and showed well-predicted domains comprising α-helices, β-sheets and unstructured tails^74^. There were more than 200 sequences in the AlphaFold multiple sequence alignment (MSA) generated for S1 and S3. Maximum likelihood (ML) analysis of MSA files revealed a close relationship of arbo-JMV S1 to NS5 from a clade comprising diverse strains of bovine viral diarrhoea viruses (BVDVs), whereas arbo-JMV S3 was closely related to NS3 clade comprising Tamana bat virus (TBV) (Fig. 4B). These observations support earlier findings demonstrating homology of S1 and S3 with the orthoflavivirus NS5 and NS3-NS2B complex, respectively^1,17^.

Notably, there were only 16–30 sequences in the MSA generated for S2 and S4, so, in contrast to the clear relationships to other orthoflaviviruses for S1 and S3, the presumed proteins from S2 and S4 have no virus proteins homologues from protein databases (Fig. 4B). This is a puzzling and very interesting finding, because although the arbo-JMV are phylogenetically related to orthoflaviviruses by several measures, the structural and nonstructural proteins come from completely different virus lineages.

## Arbo-JMV maintenance cycle in ticks and vertebrate hosts

All of the available information suggests that arbo-JMV is maintained in a natural cycle featuring alternating replication in competent arthropod vectors and susceptible vertebrate hosts. The candidate vectors include hard ticks of genera *Rhipicephalus, Amblyomma* and *Ixodes*. While arbo-JMV has also been detected in mosquitoes, transmission has not been demonstrated in this arthropod^75^. Moreover, despite arbo-JMV being associated with vertebrate hosts, such as humans, livestock and wildlife, the precise animal host or reservoir for these viruses remains speculative but the actual cycle likely involves hard ticks and vertebrate hosts.

Several examples confirm this idea, including the detection of replicating JMTV by fluorescence in-situ hybridisation (FISH) in *Amblyomma javanense* midgut and salivary glands^2^. In *A. javanense*, JMTV infection is established in the salivary gland, indicating the virus spreads from the midgut to the salivary gland after blood feeding^2^. In addition, JMTV RNA has been identified in nymphs and unfed larvae of *A. testudinarium* implying that ticks can get infected vertically through eggs from an infected adult female (transovarial transmission) as well as by feeding on infected hosts^8^. Transovarial transmission has also been shown by JMTV infection in newly hatched *Haemaphysalis longicornis* larvae^76^. Together, the findings showing arbo-JMV infection in animals as well as parasitizing ticks point to *A. javanense* as a potential JMTV reservoir and vector. However, insufficient data on vertical transmission efficiency and other potential tick species vectors hinders the implication of ticks as amplifying hosts.

Arbo-JMTV infection during tick blood feeding has been demonstrated by the detection of JMTV RNA and IgG antibodies against JMTV in humans and *I. persulcatus* collected from humans^2^. Sequencing of human-associated JMTV and tick-associated JMTV revealed up to 99.9% genome sequence homology, reinforcing this revelation^2^. Similarity in vector and host JMTV genome sequence has also been observed in rodents and parasitizing ticks. Further, the majority of JMTV has been described in engorged tick species from known animal hosts. JMTV RNA has also been detected in non-human primates, rodents, reptiles, cattle and bats^17,38^.

A similar transmission dynamic has been established for ALSV following virus detection in *I. persulcatus* and humans with a history of tick bite^28^. ALSV viral RNA and anti-ALSV antibodies have also been reported in deer and parasitizing *I. ricinus*^34^. ALSV reports in livestock include viral IgG antibodies and RNA detection in cattle and sheep by enzyme-linked immunosorbent assay (ELISA), viral neutralization test (VNT) and qRT-PCR^32^. As demonstrated for JMTV, sequencing of animal-associated ALSV and tick-associated ALSV show very high genome sequence homology suggesting tick transmission^32,34^.

**Fig. 5:**
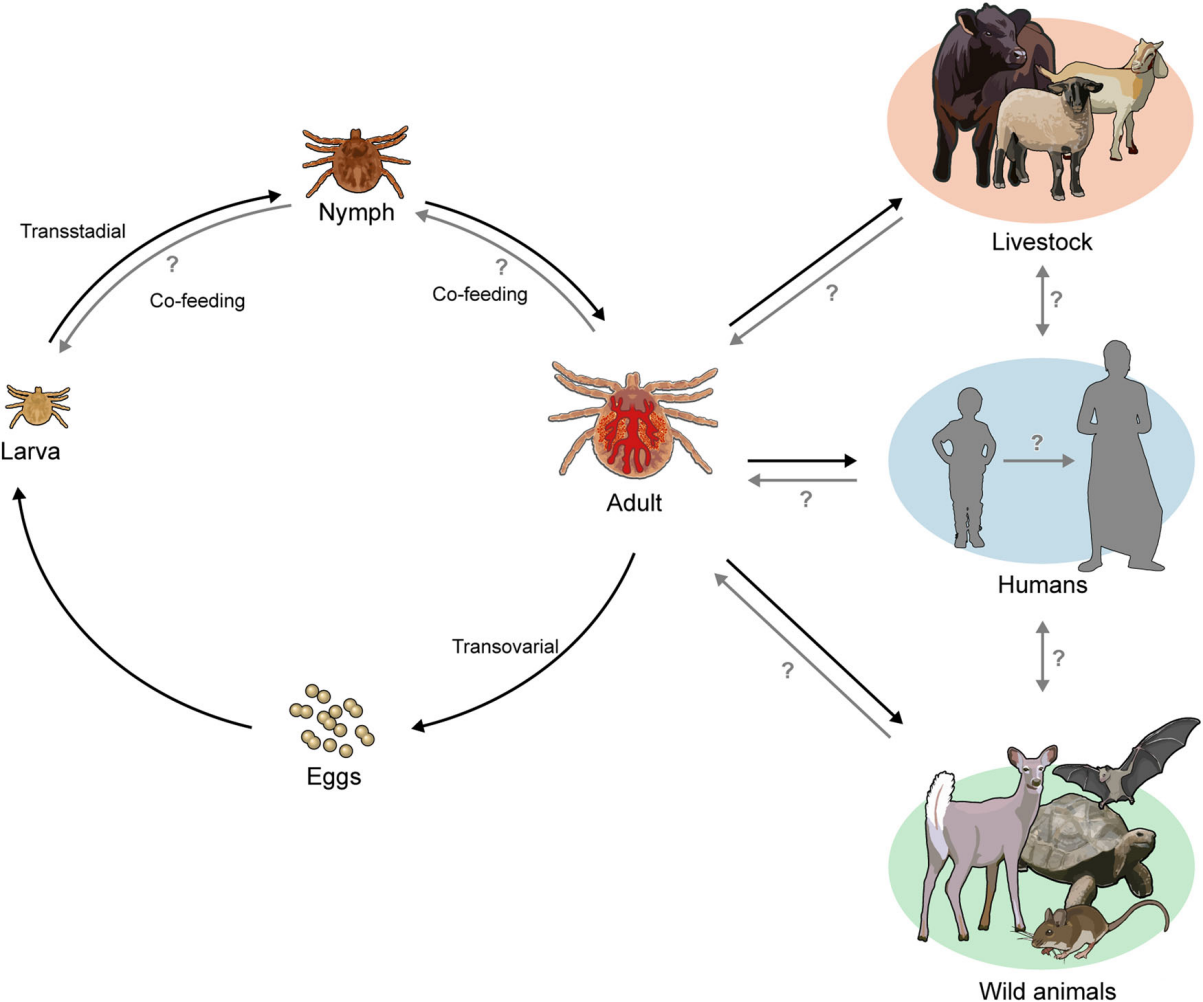
An illustration of arbo-jingmenviruses maintenance cycle in ticks and vertebrate hosts. Vertebrate hosts can get infected through a tick bite during an infectious blood-meal while ticks can get infected vertically through eggs from an infected adult female.

Generally, the definition of the host range and susceptibility of arbo-JMV is so far limited to a few viruses, including JMTV and ALSV. Some arbo-JMV represented by Sichuan tick virus (SCTV) and Mogiana tick virus (MGTV) have been reported only in ticks. Together, although sampling bias has not yet been excluded, these findings clearly infer that variants of arbo-JMV can be maintained in nature by diverse tick species and vertebrate hosts and that arbo-JMV may represent an emerging tick-borne zoonosis. The most likely arbo-JMV maintenance cycle in ticks and vertebrate hosts is illustrated in Fig. 5.

Non-viraemic transmission in ticks during co-feeding with infected ticks observed in other orthoflaviviruses such as POWV/DTV, TBEV and LIV^77–79^, or transmission in vertebrates through contact with infected vertebrates or their products such as blood and unpasteurized milk remains unestablished in arbo-JMV ^80,81^. These observations suggest that specific tick species and vertebrate hosts are key component in the maintenance system of these viruses in nature, however, transmission characteristics remain poorly investigated.

## In vitro tick and vertebrate host range

Experimental infection studies with tick and vertebrate cells show mixed outcomes but suggest a restricted in vitro host range for the arbo-JMV. JMTV isolated from *A. javanense* and *R. microplus* stably replicates in BME/CTVM23 (*R. microplus*) with the isolate showing no obvious cytopathic effects (CPE); however, virus is only detectable up to the second passage in BME26 (*R. microplus*), DH82 (*Canis familiaris*), Vero (*Cercopithecus sabaeus*) and BHK-21 (*Mesocricetus auratus*) cells^2,38,82^. ALSV isolated from humans and livestock stably replicates in Vero cells^28,32^. Stable replication of *I. persulcatus-*associated ALSV in HAE/CTVM8 (*Hyalomma anatolicum anatolicum*) and IRE/CTVM19 (*I. ricinus*) cells does not show obvious CPE^42^. In addition, ALSV proteins have been successfully expressed in human embryonic kidney (HEK293T) and liver cancer (HepG2) cells using expression vectors^30,31^. Experimental infection studies of other arbo-JMV variants, such as KITV, *Takachi virus* (TAKV) and MGTV have been sparse.

These findings indicate that in vitro arbo-JMV are likely to infect cells derived from hosts from which they were detected, however, the determinants of cellular tropism mechanism are not well defined. Furthermore, ideal cell lines for virus isolation and stable growth remain to be fully characterized.

## Evidence of arbo-JMV onward spillover

Phylogenetic analyses revealed greater genetic divergence of reptile-associated JMTV than livestock-associated JMTV. This would be consistent with JMTV in tortoise populations being subjected to relaxed selection or existing for a longer time than in livestock^38^. If JMTV existed in reptiles previous to mammals then JMTV might have spread from reptiles to mammals. Even so, evidence for and implications of onward transmission or spillover are uncertain. While blood and tissues from infected vertebrates in a population might also serve as a potential direct transmission route through contact with infected vertebrates or their products, there is no experimental data supporting this possibility. In addition, the isolated reports of JMTV RNA in wastewater and environmental samples remain difficult to evaluate^36,37^, although these findings have been taken to infer infection patterns within human and animal populations. Future studies will doubtless clarify this important area.

## Concluding remarks and future perspective

Arbo-JMV are significant new emerging tick-borne viruses. Considering the diverse potential arthropods and vertebrate hosts, it seems probable that many arbo-JMV variants exist in various geographical locations. This review article outlines the relationship between arbo-JMV and orthoflaviviruses, illustrates a potential transmission system and presents several unresolved questions. Virus identification in humans, ticks and vertebrate hosts in proximity clearly suggests public health importance. Risk may be highest among humans with close contact with livestock and animals, however, reservoir host and sources of variant emergence remains unconfirmed. Another issue is the lack of studies characterizing arbo-JMV genotypes associated with spillover. Future arbo-JMV studies designed to determine animal disease models would greatly enhance our perspective of arbo-JMV transmission and inform the development of countermeasures against these and other enigmatic arboviruses.

## Supplementary information

Supplementary information

## Data Availability

The analysed data in the current study are available within the article, Supplementary file, or public databases.
