# Supplementary material for: Strange relatives: the enigmatic arbo-jingmenviruses and orthoflaviviruses
Source: Npj Viruses. 2025 Apr 4;3:24. doi: 10.1038/s44298-025-00106-z (PMC11971299; doi:10.1038/s44298-025-00106-z)
Supplement: Supplementary file 1 — Supplementary information [file 44298_2025_106_MOESM1_ESM.pdf]

A

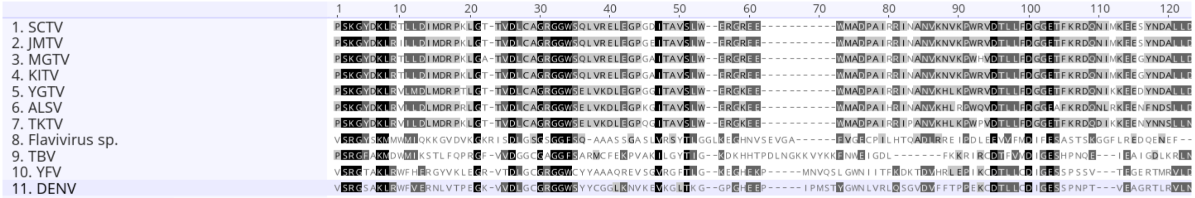

B

	SCTV	JMTV	MGTV	KITV	YGTV	ALSV	TKTV	Flavivirus sp.	TBV	YFV	DENV
SCTV		98%	96%	97%	83%	83%	82%	14%	18%	24%	27%
JMTV	98%		95%	97%	83%	83%	82%	14%	19%	24%	28%
MGTV	96%	95%		98%	82%	82%	82%	14%	18%	24%	28%
KITV	97%	97%	98%		84%	83%	83%	14%	19%	24%	28%
YGTV	83%	83%	82%	84%		85%	86%	15%	19%	27%	28%
ALSV	83%	83%	82%	83%	85%		86%	15%	18%	25%	28%
TKTV	82%	82%	82%	83%	86%	86%		15%	20%	25%	28%
Flavivirus sp.	14%	14%	14%	14%	15%	15%	15%		24%	27%	24%
TBV	18%	19%	18%	19%	19%	18%	20%	24%		32%	35%
YFV	24%	24%	24%	24%	27%	25%	25%	27%	32%		58%
DENV	27%	28%	28%	28%	28%	28%	28%	24%	35%	58%	

Supplementary Figure 1. Comparison of arbo-jingmenviruses and orthoflaviruses methyltransferase (MTase) amino acid sequences. A, MTase gene of arbo-jingmenviruses and representative of closely associated orthoflaviruses. **B,** MTase gene distance matrix showing representative orthoflaviruses and arbo-jingmenviruses.

Supplementary Table 1 Detection and geographic distribution of arbo-jingmenviruses.

Arbo-jingmenvirus (Abbr.)	arthropod species	Host	Antibodies Detected	Region	Country	Reference
Jingmen tick virus (JMTV)	*H. Longicornis, I. ovatus, I. acutitarsus*	Goat, Panda	No	Sichuan	China	84
	R. microplus	Cattle	No	Yunnan	China	85
	Unknown*	Cattle, Sheep, Human	Cattle, Sheep	Hubei	China	86
	I. persulcatus	Host seeking	No	Heilongjiang	China	87
	R. microplus	Cattle	No	Guizhou	China	45
	R. microplus, A. variegatum, Rhipicephalus spp.	Cattle, Sheep	No	Efoulan, Akam-Engali, Akena, Ngoulemakong	Cameroon	9
	I. persulcatus, D. silvarum, H. concinna	Human	No	Heilongjiang	China	29
	Rhipicephalus	Cattle, Goat	No	Arua, Nakaseke	Uganda	88
	R. sanguineus	Canine, Cattle	No	Hainan	China	89
	I. simplex, R.bursa	Sheep, Bat	No	Izmir, Ankara	Turkey	90
	Unknown*	Unknown‡	No	Nagpur	India	36
	R. appendiculatus, R. evertsi evertsi, Hy. Truncatum, Amblyomma sp. *A. sparsum, A. nuttalli*	Goat, Sheep, Tortotise, Cattle	No	Baringo, Kajiado	Kenya	38
	Unknown*	Cattle, Soil metagenome	No	Hainan	China	37
	A. testudinarium	Host seeking	No	Kanto	Japan	91
	R. microplus, A. variegatum, R. geigyi	Unknown	No	Boola	Guinea	92
	R. bursa, R. turanicus	Sheep, Cattle	No	Sanlıurfa Antalya	Turkey	93
	H. longicornis	Cattle	No	Yunnan	China	78
	I. ricinus	Unknown‡	No	Moscow	Russia	65
	H. Longicornis, R. microplus, I. sinensis	Dog, Cattle, Sheep	No	Henan, Guizhou, Yunnan, Anhui, Shandong	China	94
	A. testudinarium, H. longicornis	Boars	No	Fujian	China	95
	Rhipicephalus spp.	Elephant, Cattle, Lion Hartebeest, Wild Dog	No	Laikipia, Meru, Samburu, Marsabit, Isiolo	Kenya	96
	A. testudinarium	Unknown‡	No	Nagasaki, Ehime	Japan	8
	H. longicornis, R. microplus	Goat, Cattle	No	Hubei	China	97
	R. bursa	Sheep, Goat	No	Tulcea	Romania	98

	R. microplus	Cattle	Cattle	Yunnan	China	[99]
	Ae. albopictus	Unknown‡	No	Rimini	Italy	[77]
	I. ricinus	Unknown‡	No	Belgrade	Serbia	[100]
	Unknown*	Rodents	No	Xinjiang	China	[101]
	R. microplus, A. variegatum	Cattle	No	Guadeloupe, Martinique	France	[7]
	R. microplus	Cattle		Antioquia	Colombia	[102]
	H. hystricis, R. microplus	Bats, Rodents, Cattle	No	Zhejiang, Henan, Guizhou	China	[103]
	Unknown*	Mouse	No	Pennsylvania	USA	[4]
	A. javanense, I. persulcatus	Human, Pangolins	Human	Guangxi	China	[2]
	R. microplus, Amblyomma testudinarium, I. ricinus	Bat, Human	Human	Guadeloupe, Martinique, Alsace	France, Laos, Cambodia	[104]
	R. bursa, R. turanicus, Hy. marginatum, H. parva, R. Sanguineus sensu lato, *H. inermis*	Dog, Sheep, Goat, Cattle	No	Van, Mersin, Kirklareli, Tekirdag	Turkey	[3]
	I. persulcatus, H. longicornis, H. concinna, D. nuttalli	Unknown‡		Heilongjiang	China	[105]
	R. bursa, Hy. marginatum, R. sanguineus	Cattle, Sheep	No	Corsica	France	[10]
	R. sanguineus	Canine, Bovine		Danzhou, Lingao	China	[106]
	R. microplus	Cattle		Guarapuava, Lages, Manoel Ribas, Ronda Alta	Brazil	[107]
	Unknown*	Human	No	Senik, Negrovc, Rahovec	Kosovo	[108]
	Unknown*	Monkey	No	Kibale	Uganda	[17]
	H. longicornis	Unknown‡	No	Macheng, Nanzhang	China	[109]
	R. microplus	Cattle	No	Loudi	China	[46]
	D. silvarum, H. longicornis	Wild boars	No	Jiangxi	China	[110]
	R. microplus, D. nitens, A. dissimile	Bovine, Iguana	No	Córdoba, Cesar	Colombia	[111]
	R. microplus, H. longicornis, H. campanulate, H. flava, I. sinensis, R. sanguineus, I. granulatus, Armigeres sp.	Cattle, Dog, Goat, Hedgehog Badger, Wild goat	Cattle	Jingmen, Hubei, Zhejiang	China	[1]
Alongshan virus (ALSV)	*Ixodes* spp.	Deer	Goat, Sheep, Deer, Horse	Lower Saxony	Germany	[34]
	I. persulcatus	Unknown‡		Heilongjiang	China	[112]
	H. longicornis	Unknown‡		Liaoning	China	[113]
	I. persulcatus	Unknown‡	No	Chelyabinsk	Russia	[42]

	I. persulcatus	Unknown‡		Karelia Chelyabinsk	Russia	[40]
	H. longicornis	Unknown‡		Macheng, Nanzhang	China	[109]
	H. longicornis	Unknown‡	No	Liaoning	China	[114]
	I. persulcatus	Unknown‡	No	Mongolia	China	[115]
	D. reticulatus, I. Ricinus, H. concinna, D. nuttalli, I. persulcatus	Unknown‡		Ulyanovsk, Tatarstan, Tuva, Kaliningrad, Altai, Chelyabinsk, Karelia,	Russia	[39]
	I. persulcatus, Cx. Tritaeniorhynchus An. yatsushiroensis	Human	Human	Mongolia, Heilongjiang, Jilin	China	[28]
	Unknown	Sheep, Cattle	Sheep, Cattle	Mongolia	China	[32]
	I. ricinus	Unknown‡	No	Kotka archipelago	Finland	[33]
	I. persulcatus	Unknown‡	No	Khakassia, Tyva, Irkutsk Oblast, Transbaikal	Siberia	[116]
	R. microplus	Cattle	No	Aripo, Centeno, Cedros	Trinidad and Tobago	[6]
Baishan forest tick virus (BSFTV)	*D. nuttalli*	Unknown‡	No	Xinjiang	China	[117]
Yanggou tick virus (YGTV)	*D. reticulatus, D. marginatus, I. persulcatus*	Unknown‡	No	Chelyabinsk	Russia	[42]
	D. nuttalli, D. marginatus	Cattle		Tuva, Altai	Russia	[39]
	I. persulcatus	Unknown‡	No	Baishan	China	[118]
	D. nuttalli	Unknown‡	No	Xinjiang	China	[117]
Mogiana tick virus (MGTV)	*H. longicornis, I. ovatus, I. acutitarsus*	Goat, Panda	No	Sichuan	China	[84]
	A. testudinarium	Buffalo	No	Yunnan	China	[119]
	R. microplus	Cattle		Antioquia	Colombia	[102]
	R. microplus	Cattle	No	Uberlândia	Brazil	[5]
	R. microplus	Cattle	No	São Paulo	Brazil	[47]
	R. microplus	Cattle		São Paulo	Brazil	[120]
Kindia tick virus (KITV)	*R. geigyi, R. annulatus, R. decoloratus*	Livestock	No	Kindia	Guinea	[121]
	R. geigyi	Cattle	No	Kindia	Guinea	[122]
Xinjiang tick virus 1 (XJTV)	*Hy. asiaticum*	Sheep	No	Xinjiang	China	[123]

Takachi virus (TAKV)	*H. formosensis*	Unknown[‡]	No	Ehime	Japan	8
Sichuan tick virus (SCTV)	*I. acutitarsus, I. ovatus, H. longicornis*	Panda, Goat	No	Sichuan	China	84
Newport tick virus (NTV)	*I. holocyclus*	Unknown[‡]	No	Kiola	Australia	48
Histiostoma Jingmenvirus (HJMV)	*Histiostoma* sp.	Unknown[‡]	No	Solling	Germany	27

*: Vector not surveyed; [‡]: Tick and and Mite collected from the environment and not from the vertebrate host; *R.*: *Rhipicephalus; A.*: *Amblyomma; I.: Ixodes; H.: Haemaphysalis; D.: Dermacentor; Hy*.: *Hyalomma: Ae.: Aedes; Cx.: Culex; An.: Anopheles.*

84. Huang, L. *et al.* Identification of novel Jingmen tick virus from parasitic ticks fed on a giant panda and goats in Sichuan Province, southwestern China. *Front Microbiol* **14**, (2023).

85. Qu, L. *et al.* Identification and Characterization of Jingmen Tick Virus in Ticks from Yunnan Imported Cattle. *Vector-Borne and Zoonotic Diseases* **23**, 298–302 (2023).

86. Zhang, X**.**, Li, B., Wang, J., Sun, H., Zheng, Y., Luo, X. A preliminary study of Jingmen tick virus infection in human and domestic animals in Hubei, 2022. *Dis. Surveill.* **38**, 537–542 (2023).

87. Qin, T., Shi, M., Zhang, M., Liu, Z., Feng, H. & Sun, Y. Diversity of RNA viruses of three dominant tick species in North China. *Front. Vet. Sci.* **9**, 1057977 (2023).

88. Atim, S. A. *et al.* Ticks; a reservoir for virus emergence at the human-livestock interface in Uganda. *bioRxiv* (2023).

89. Wang, G. *et al.* Genomic and phylogenetic profiling of RNA of tick-borne arboviruses in Hainan Island, China. *Microbes Infect.* **26**, 105218 (2024).

90. Ergunay, K. *et al.* Impact of nanopore-based metagenome sequencing on tick-borne virus detection. *Front Microbiol* **14**, (2023).

91. Matsumura, R., Kobayashi, D., Itoyama, K. & Isawa, H. First detection of the Jingmen tick virus in Amblyomma testudinarium ticks from the Kanto district, Japan. *Jpn. J. Infect. Dis.* ***77**, 174–177 (2024)*.

92. Skripnichenko, D. D. *et al*. Jingmen tick virus in Guinea. https://www.ncbi.nlm.nih.gov/nuccore/PP779174.

93. Dinçer, E. *et al.* Several Tick-Borne Pathogenic Viruses in Circulation in Anatolia, Turkey. *Vector-Borne and Zoonotic Diseases* **22**, 148–158 (2022).

94. Pang, Z. *et al.* Geographical distribution and phylogenetic analysis of Jingmen tick virus in China. *iScience* **25**, (2022).

95. Zhang, Y. *et al.* Identification of Jingmen tick virus (JMTV) in Amblyomma testudinarium from Fujian Province, southeastern China. *Parasit Vectors* **15**, (2022).

96. Ergunay, K. *et al.* Metagenomic Investigation of Ticks From Kenyan Wildlife Reveals Diverse Microbial Pathogens and New Country Pathogen Records. *Front Microbiol* **13**, (2022).

97. Xu, L. *et al.* Tick virome diversity in Hubei Province, China, and the influence of host ecology. *Virus Evol* **7**, (2021).

98. Bratuleanu, B. E. *et al.* The virome of Rhipicephalus, Dermacentor and Haemaphysalis ticks from Eastern Romania includes novel viruses with potential relevance for public health. *Transbound Emerg Dis* **69**, 1387–1403 (2022).

99. Shi, J., Shen, S., Wu, H., Zhang, Y. & Deng, F. Metagenomic Profiling of Viruses Associated with Rhipicephalus microplus Ticks in Yunnan Province, China. *Virol Sin* **36**, 623–635 (2021).

100. Zhang, Y. *et al.* Depicting the RNA Virome of Hematophagous Arthropods from Belgrade, Serbia. *Viruses* **12**, (2020).

101. Yu, Z. M. *et al.* Identification and characterization of Jingmen tick virus in rodents from Xinjiang, China. *Infect. Genet. Evol.* **84**, 104411 (2020).

102. Gómez, G. F., Isaza, J. P., Segura, J. A., Alzate, J. F. & Gutiérrez, L. A. Metatranscriptomic virome assessment of Rhipicephalus microplus from Colombia. *Ticks Tick Borne Dis* **11**, (2020).

103. Guo, J. J. *et al.* Diversity and circulation of Jingmen tick virus in ticks and mammals. *Virus Evol* **6**, (2020).

104. Temmam, S. *et al.* Insights into the Host Range, Genetic Diversity , and Geographical Distribution of Jingmenviruses. *Ecological and Evolutionary Science* **4**, 1–13 (2019).

105. Meng, F. *et al.* Virome analysis of tick-borne viruses in Heilongjiang Province, China. *Ticks Tick Borne Dis* **10**, 412–420 (2019).

106. Wang, G. *et al.* Genomic and phylogenetic profiling of RNA of tick-borne arboviruses in Hainan Island, China. *Microbes Infect* **26**, 105218 (2024).

107. Souza de, W. M. *et al.* Viral diversity of Rhipicephalus microplus parasitizing cattle in southern Brazil. *Sci Rep* **8**, (2018).

108. Emmerich, P. *et al.* Viral metagenomics, genetic and evolutionary characteristics of Crimean-Congo hemorrhagic fever orthonairovirus in humans, Kosovo. *Infection, Genetics and Evolution* **65**, 6–11 (2018).

109. Xiao, J. *et al.* Viromes of Haemaphysalis longicornis reveal different viral abundance and diversity in free and engorged ticks. *Virol Sin* **39**, 194–204 (2024).

110. Liu, Z. *et al.* Identification and phylogenetic analysis of Jingmen tick virus in Jiangxi Province, China. *Front Vet Sci* **11**, (2024).

111. López, Y. *et al.* Hard ticks (Ixodida: Ixodidae) in the Colombian Caribbean harbor the Jingmen tick virus: an emerging arbovirus of public health concern. *Parasit Vectors* **17**, 1–7 (2024).

112. Cai, X. *et al.* Virome analysis of ticks and tick-borne viruses in Heilongjiang and Jilin Provinces, China. *Virus Res* **323**, (2023).

113. Bai, Y. *et al.* Analysis of the diversity of tick-borne viruses at the border areas in Liaoning Province, China. *Front Microbiol* **14**, (2023).

114. Hu, H. X. Alongshan virus isolate Liaoning glycoprotein gene, complete cds - Nucleotide - NCBI. https://www.ncbi.nlm.nih.gov/nuccore/MZ676705.1 (2022).

115. Liu, Z. *et al.* Extensive diversity of RNA viruses in ticks revealed by metagenomics in northeastern China. *PLoS Negl Trop Dis* **16**, (2022).

116. Kartashov, M. Y. *et al.* Prevalence and genetic diversity of the Alongshan virus (Flaviviridae) circulating in ticks in the south of Eastern Siberia. *Vopr Virusol* **69**, 151–161 (2024).

117. Shen, S. *et al.* Yanggou tick virus strain YG NS5-like protein gene, complete cds - Nucleotide - NCBI. https://www.ncbi.nlm.nih.gov/nuccore/MH688529.1 (2019).

118. Wang, R. *et al.* Metatranscriptomics Reveals the RNA Virome of Ixodes Persulcatus in the China–North Korea Border, 2017. *Viruses* **16**, 62 (2023).

119. Xu, X. *et al.* Full-length genome sequence of segmented RNA virus from ticks was obtained using small RNA sequencing data. *BMC Genomics* **21**, 1–8 (2020).

120. Villa, E. C., Maruyama, S. R., de Miranda-Santos, I. K. F., Palacios, G. & Ladner, J. T. Complete coding genome Sequence for mogiana tick virus, a jingmenvirus isolated from ticks in Brazil. *Genome Announc* **5**, 17–18 (2017).

121. Kartashov, M. Y. *et al.* Molecular and genetic characteristics of the multicomponent flavi-like Kindia tick virus (Flaviviridae) found in ixodes ticks on the territory of the Republic of Guinea. *Vopr Virusol* **67**, 487–495 (2022).

122. Ternovoi, V. A. *et al.* Complete coding genome sequence for a novel multicomponent Kindia tick virus detected from ticks collected in Guinea. *bioRxiv* (2020).

123. Yang, Z. *et al.* Virome diversity of ticks feeding on domestic mammals in China. *Virol Sin* **38**, 208–221 (2023).
